# Synchronous ipsilateral clear cell renal cell carcinoma and upper tract urothelial carcinoma complicated by ureteral stone: a case report

**DOI:** 10.1093/jscr/rjag1031

**Published:** 2026-11-10

**Authors:** Khanh N Tran, Thao N P Phan, Liem T Ngo, Vinh L Le, Linh H Tran, Kha C L Bui

**Affiliations:** Department of Urology B, Hue Central Hospital, 16 Le Loi Street, Thuan Hoa Ward, Hue 49135, Vietnam; Department of Urology B, Hue Central Hospital, 16 Le Loi Street, Thuan Hoa Ward, Hue 49135, Vietnam; Department of Urology B, Hue Central Hospital, 16 Le Loi Street, Thuan Hoa Ward, Hue 49135, Vietnam; Department of Urology B, Hue Central Hospital, 16 Le Loi Street, Thuan Hoa Ward, Hue 49135, Vietnam; Department of Urology B, Hue Central Hospital, 16 Le Loi Street, Thuan Hoa Ward, Hue 49135, Vietnam; Department of Urology B, Hue Central Hospital, 16 Le Loi Street, Thuan Hoa Ward, Hue 49135, Vietnam

**Keywords:** clear cell renal cell carcinoma, upper tract urothelial carcinoma, synchronous malignancies, ureteral calculus, hydronephrosis, radical nephroureterectomy

## Abstract

Synchronous renal cell carcinoma (RCC) and upper tract urothelial carcinoma (UTUC) in one renal unit are rare and diagnostically challenging. A 52-year-old man with flank pain and hematuria had an enhancing renal mass, hydronephrosis, ureteral wall thickening, impaired excretion, and an obstructing ureteral stone. Magnetic resonance imaging suggested synchronous RCC and UTUC. Laparoscopic radical nephroureterectomy with bladder cuff excision and left renal hilar lymph-node sampling revealed WHO/ISUP grade 2 clear cell RCC (pT1aN0) and low-grade invasive UTUC (pT1N0). At 6 months, imaging and cystoscopic biopsies showed no recurrence. Imaging raised suspicion of synchronous malignancies, while histopathology confirmed both diagnoses and demonstrated less extensive UTUC than suggested radiologically.

## Introduction

Renal cell carcinoma (RCC) and upper tract urothelial carcinoma (UTUC) differ in origin and treatment [1–3]. Only limited cases of synchronous ipsilateral RCC and UTUC have been reported, involving clear cell, papillary, and chromophobe RCC [4–6]. Distinguishing two primary tumors from metastatic disease is important because it directly affects staging, treatment, and surveillance [3, 7, 8].

An obstructing stone can cause hematuria, hydronephrosis, urothelial thickening, and impaired excretion, obscuring UTUC. We report synchronous ccRCC and UTUC with an obstructing ureteral stone.

## Case presentation

A 52-year-old man presented with left flank pain radiating to the lower abdomen and groin and mild gross hematuria. He had no fever, lower urinary tract symptoms, weight loss, smoking history, or occupational carcinogen exposure. His medical and surgical histories were unremarkable, and his Eastern Cooperative Oncology Group performance status was 1. Hemoglobin was 12.8 g/dl, serum creatinine 114 μmol/l, and estimated glomerular filtration rate (eGFR) 63.35 ml/min/1.73 m^2^. Urinalysis showed hematuria without pyuria, and urine culture was negative.

Contrast-enhanced computed tomography (CT) showed an 18 × 13-mm, well-defined, avidly enhancing mass in the midportion of the left kidney. The collecting system had grade II hydronephrosis, proximal ureteral dilatation to 12 mm, an 11 × 7-mm upper-ureteral calculus, and irregular thickening and fat stranding of the renal pelvis and proximal ureter (Fig. 1A). Delayed images at 20 min showed impaired left renal enhancement without contrast excretion, whereas the right kidney excreted normally (Fig. 1B).

**Figure 1:**
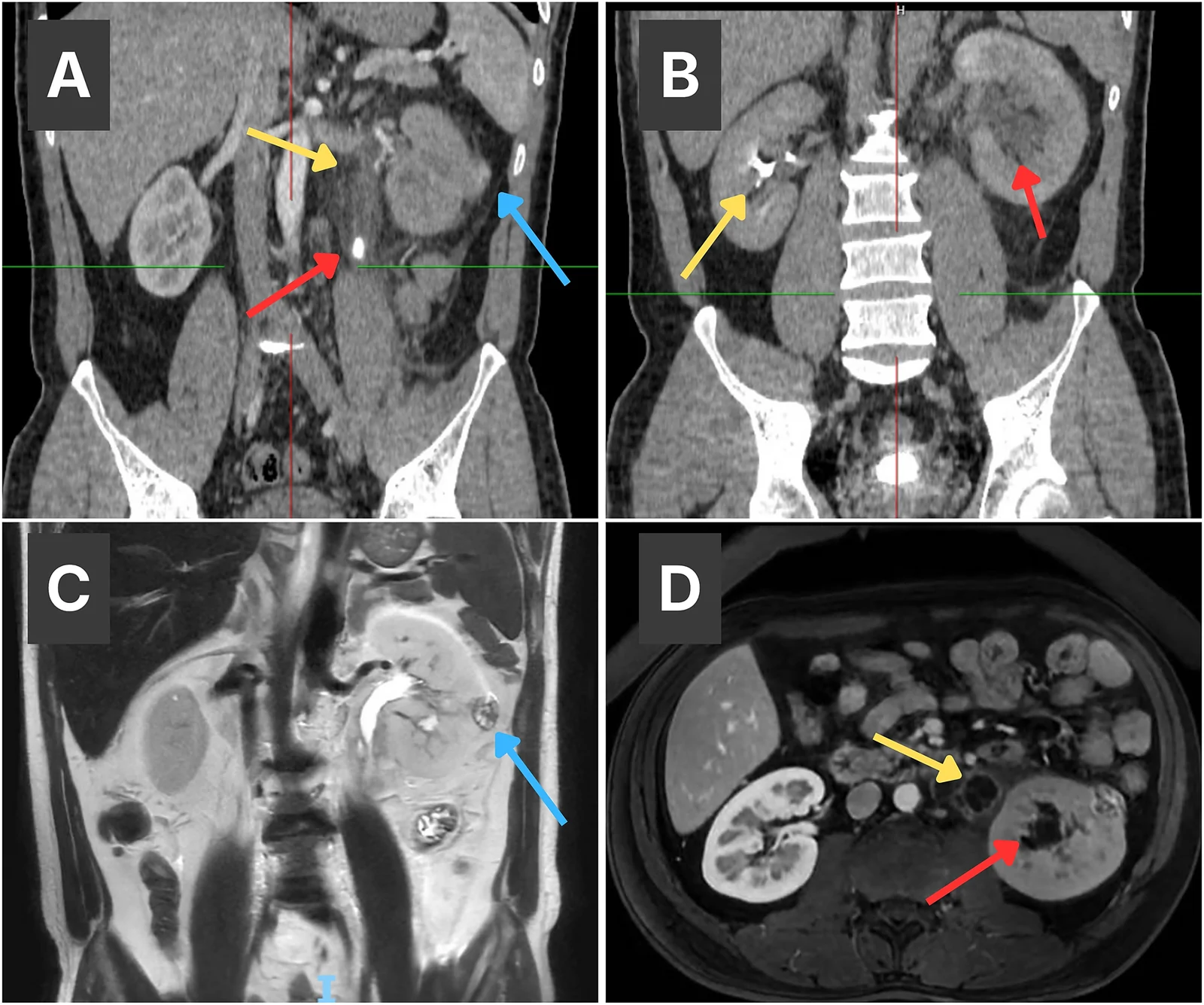
Preoperative imaging of the left renal and upper urinary tract lesions. (A) Contrast-enhanced CT in the coronal plane demonstrates an avidly enhancing 18 × 13-mm mass in the midportion of the left kidney (blue arrow), irregular thickening and dilatation of the proximal ureter (yellow arrow), and an 11 × 7-mm upper ureteral stone (red arrow). (B) Delayed-phase CT demonstrates left hydronephrosis and impaired renal excretion, with no contrast opacification of the left collecting system (red arrow), whereas the right kidney shows preserved excretion (yellow arrow). (C) Contrast-enhanced MRI demonstrates the enhancing renal parenchymal mass (blue arrow). (D) MRI demonstrates irregular thickening and enhancement of the left renal pelvis (red arrow) and proximal ureter with surrounding periureteral soft-tissue changes (yellow arrow).

Because impaired excretion limited upper-tract assessment on CT, contrast-enhanced magnetic resonance imaging (MRI) was performed. MRI showed an ~5-cm segment of irregularly enhancing renal pelvic and proximal ureteral wall thickening, suspicious for periureteral invasion. A separate 17 × 18-mm renal parenchymal mass showed early enhancement and delayed washout (Fig. 1C, D), raising suspicion of synchronous RCC and UTUC. Staging CT showed no distant metastasis. Voided urine cytology was negative. Preoperative cystoscopy revealed no suspicious bladder lesions; therefore, no biopsy was performed. Left ureteroscopy was attempted but halted by the obstructing 11 × 7-mm proximal ureteral stone, precluding biopsy and selective cytology. Staged lithotripsy followed by repeat ureteroscopy was considered but not pursued because of concerns regarding ureteral injury and MRI-suspected invasive UTUC. Consequently, the absence of preoperative histological confirmation limited diagnostic certainty and risk stratification.

Given MRI-suspected invasive UTUC, laparoscopic radical nephroureterectomy with bladder cuff excision and hilar node sampling was performed after informal interdisciplinary discussion. Kidney-sparing alternatives were discussed with the patient, who consented to radical surgery. Differential renal function was not formally assessed. No gross invasion of adjacent structures was observed. The specimen contained a circumscribed whitish mid-renal mass, dilatation of the renal pelvis and proximal ureter, and a 10 × 8-mm proximal ureteral stone (Fig. 2). Recovery was uncomplicated, and the patient was discharged on postoperative day 6.

**Figure 2:**
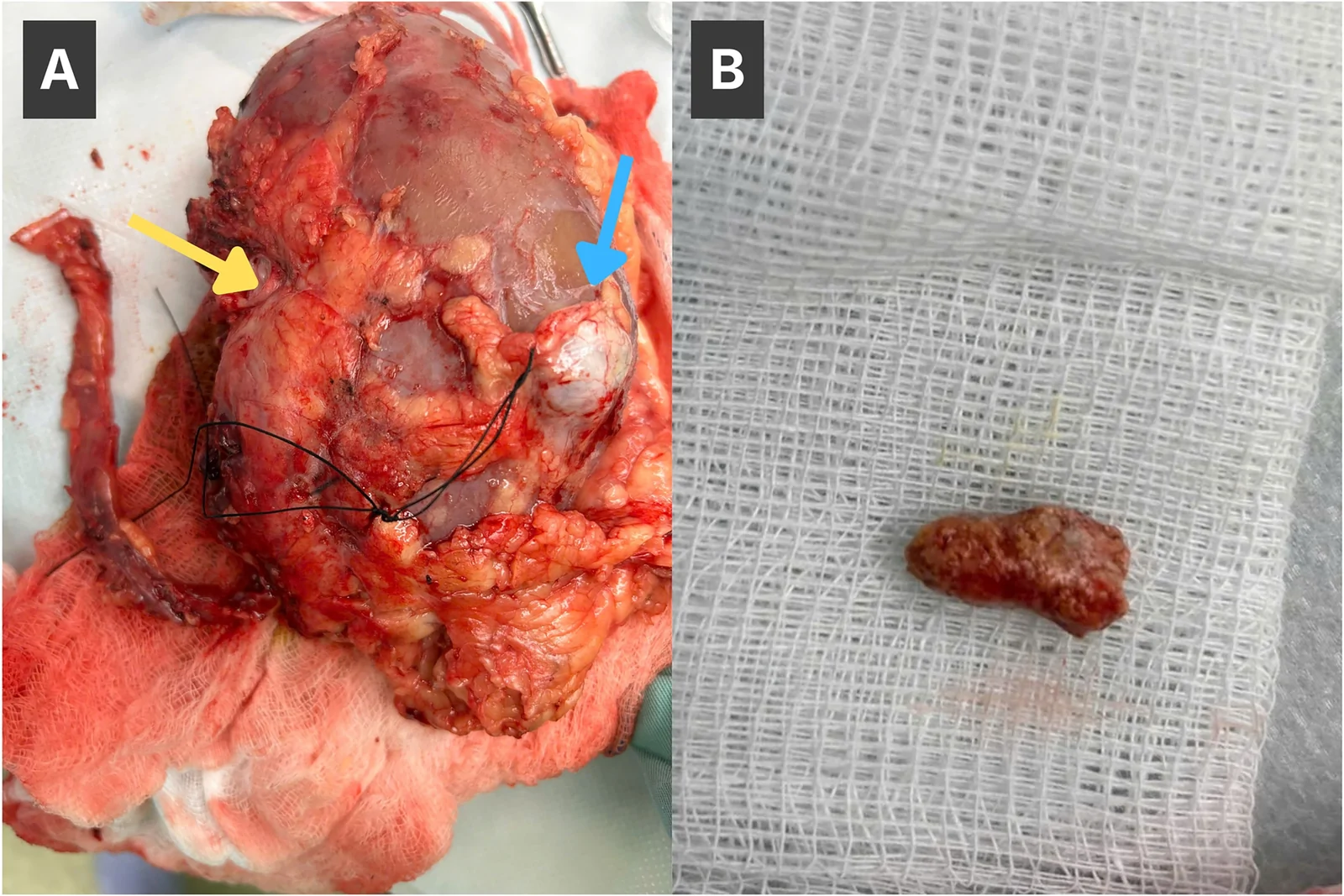
Surgical specimen and ipsilateral ureteral stone. (A) Gross nephroureterectomy specimen showing a well-circumscribed whitish mass in the midportion of the left kidney (blue arrow) and dilatation of the renal pelvis and proximal ureter (yellow arrow). (B) the ureteral stone, measuring ~10 × 8 mm, retrieved from the proximal left ureter.

Histopathology confirmed a 2-cm WHO/ISUP grade 2 ccRCC (pT1a) and a 2.8 × 2.2-cm low-grade urothelial carcinoma at the ureteropelvic junction, invading the lamina propria (pT1) (Fig. 3). No concomitant carcinoma in situ was identified. The urothelial tumor was adjacent to an impacted calculus, with marked inflammation and fibrosis contributing to MRI-suspected periureteral invasion. No muscular, lymphovascular, or perineural invasion was identified. Surgical margins were negative. Both sampled hilar lymph nodes were negative (0/2; pN0), although nodal assessment was limited.

**Figure 3:**
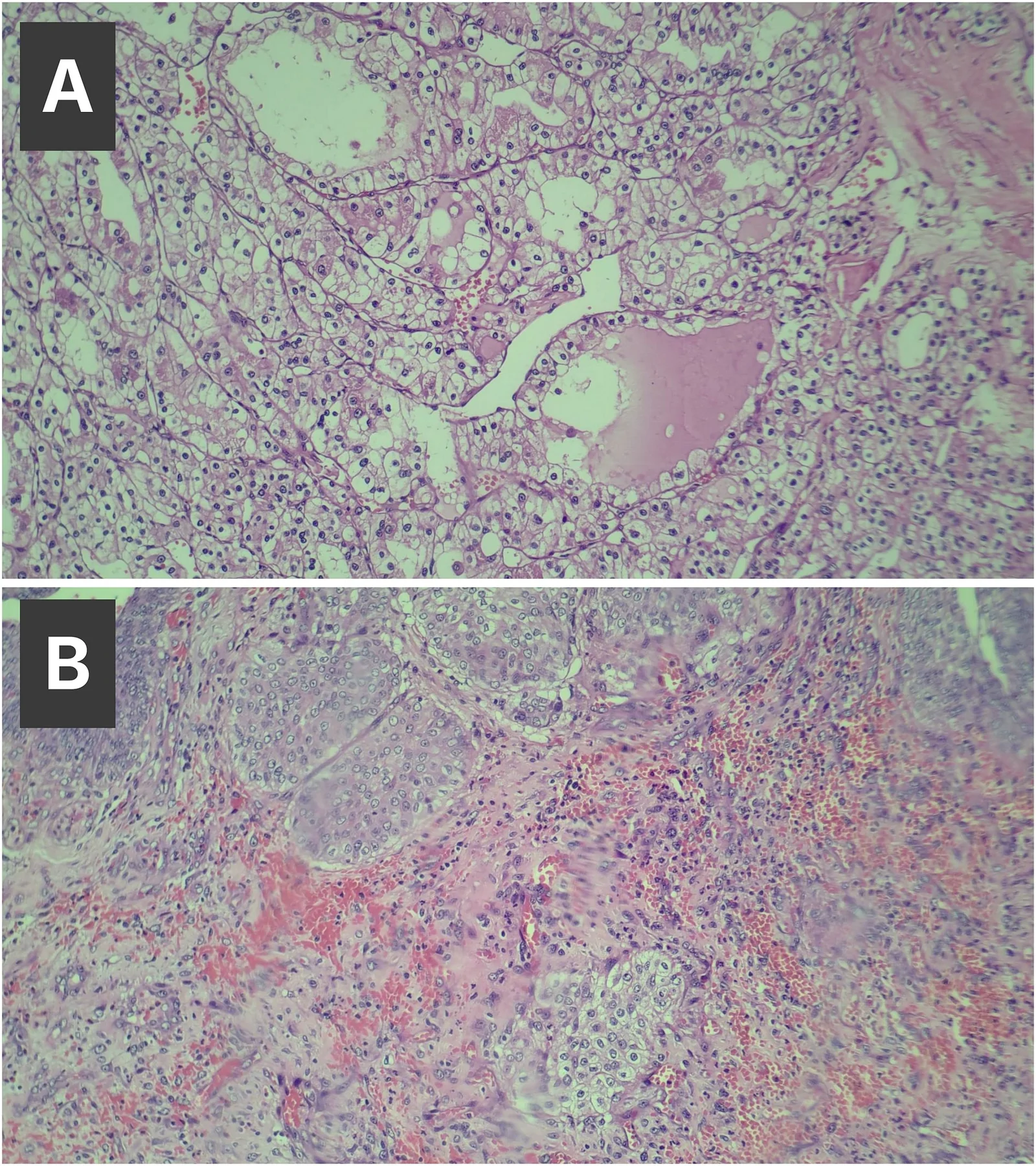
Histopathological features of the synchronous renal and urothelial tumors. (A) Clear cell RCC, WHO/ISUP grade 2, composed of polygonal cells with clear cytoplasm arranged in nests separated by a delicate vascular network (hematoxylin and eosin staining, ×400). (B) Invasive low-grade urothelial carcinoma of the upper urinary tract showing atypical urothelial cells arranged in nests and small clusters with invasion into the subepithelial connective tissue (hematoxylin and eosin staining, ×400).

Neither adjuvant systemic therapy nor postoperative intravesical chemotherapy was administered. At 3 months, cystoscopy showed no bladder tumor. At 6 months, the patient was clinically well; serum creatinine was 94.2 μmol/L and eGFR 79.78 ml/min/1.73 m^2^. CT showed no recurrence or metastasis but identified focal bladder-wall thickening near the previous cuff site, considered inflammatory or fibrotic (Fig. 4A). Cystoscopy showed no tumor, and biopsies from the cuff site and other bladder locations were negative (Fig. 4B, C).

**Figure 4:**
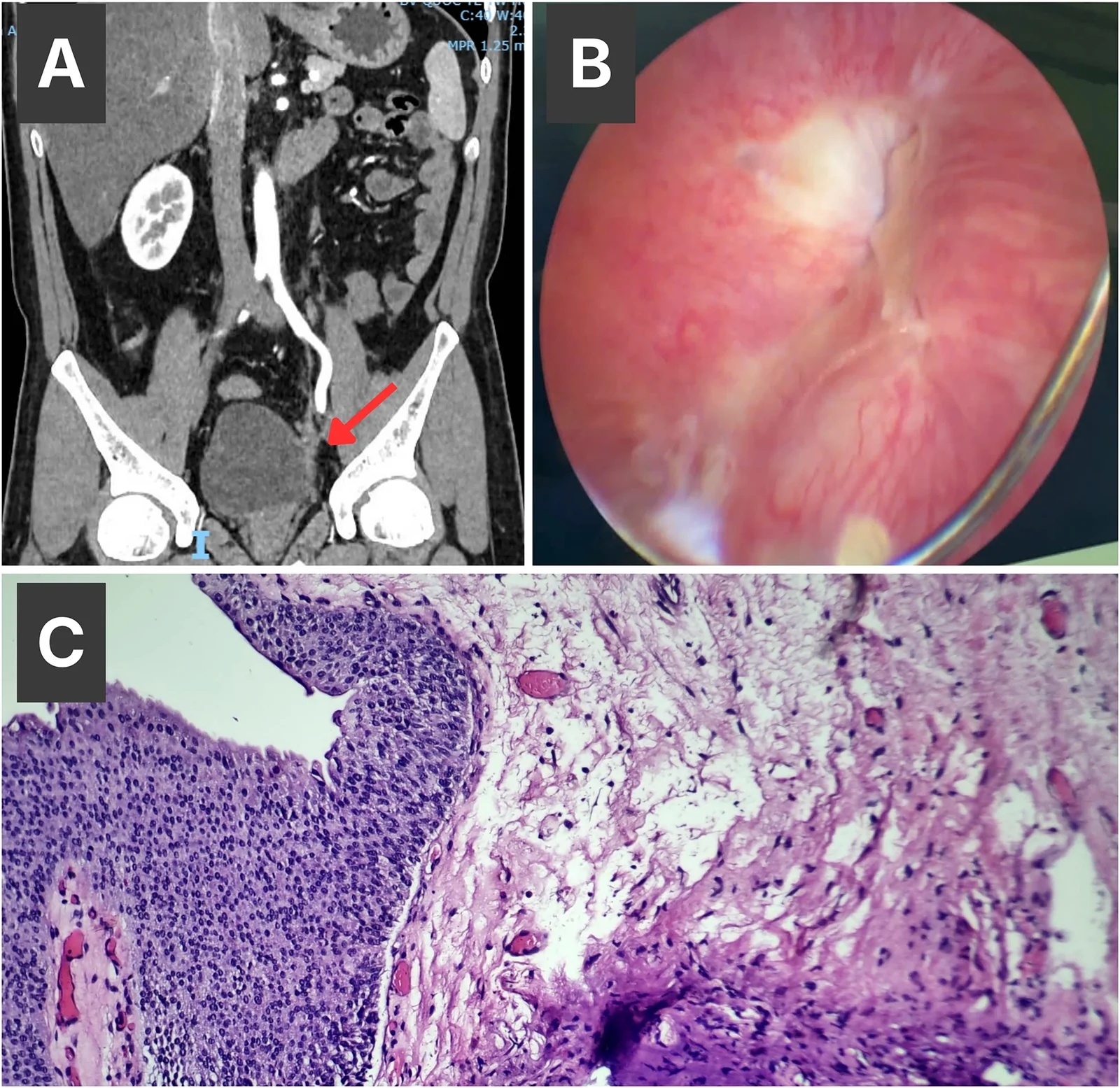
Six-month postoperative surveillance findings. (A) Contrast-enhanced CT demonstrates focal bladder-wall thickening at the 3–4 o’clock position near the previous bladder cuff excision site (red arrow), without evidence of local recurrence or distant metastasis. (B) Cystoscopic examination shows the healed bladder cuff excision site without a visible recurrent tumor. (C) Histopathological examination of a representative bladder biopsy shows benign urothelium without evidence of malignancy at the previous bladder cuff site (hematoxylin and eosin staining, ×400).

## Discussion

A literature review identified 56 patients with RCC and urothelial carcinoma of the ipsilateral renal pelvis or ureter [4]. In a cohort of 61 454 RCC patients, 704 had synchronous urothelial carcinoma, including 138 with UTUC [7]. Clear cell RCC predominated [4–6].

The principal diagnostic challenge was the obstructing stone. CT urography is preferred for UTUC [3, 9]. However, absent contrast excretion prevented adequate assessment of the collecting system. Furthermore, obstruction and inflammation can cause urothelial thickening and periureteral stranding that mimic malignancy [9, 10]. MRI demonstrated that the upper-tract abnormality was anatomically separate from the renal mass. Its greater radiological than pathological extent, with final pT1 disease, suggests that inflammation contributed to the imaging appearance.

Stones are associated with UTUC, although causality remains unproven [11, 12]. Unlike a previous report involving a contralateral staghorn calculus [13], the stone in our patient obstructed the tumor-bearing ureter, potentially masking UTUC. In another report, synchronous urothelial carcinoma was diagnosed only after nephrectomy [4]. Suspicious urothelial abnormalities therefore warrant evaluation even when a calculus offers an alternative explanation.

The 18-mm renal mass alone favored partial nephrectomy [8]. However, extensive irregular urothelial enhancement and periureteral changes on MRI raised concern for invasive UTUC, favoring radical nephroureterectomy [3]. Stone-related inflammation may have mimicked invasion, as suggested by the final pT1 stage. The absence of preoperative tissue confirmation limited risk stratification [3].

Postoperative intravesical chemotherapy was not administered, without a documented contraindication or rationale, representing a limitation of perioperative management despite guideline recommendations [3]. Given the 2.8-cm tumor and preoperative imaging suspicion of invasion, an intensified EAU high-risk surveillance schedule was adopted despite low-grade pT1 pathology. Following negative 3-month cystoscopy, surveillance comprises cystoscopy and urinary cytology every 6 months for 2 years, then annually through year 5; CT urography and chest CT every 6 months for 2 years, then annually.

Despite negative preoperative cystoscopy, the absence of upper-tract histological confirmation limited diagnostic certainty and evaluation of kidney-sparing alternatives, particularly given the potential for stone-related inflammation to mimic tumor invasion [3].

## Conclusion

An obstructing ureteral calculus may obscure UTUC accompanying RCC. Imaging raised suspicion but overestimated UTUC extent; histopathology confirmed both malignancies. Suspicious urothelial abnormalities warrant evaluation despite a concurrent calculus. Six-month follow-up cannot establish durable oncological control.
